# Reliability of impedance spectroscopy versus digital radiograph and ICDAS-II in occlusal caries detection: a prospective clinical trial

**DOI:** 10.1038/s41598-024-66627-4

**Published:** 2024-07-17

**Authors:** Amr R. AbdELkader, Shereen Hafez Ibrahim, Olfat Elsayed Hassanein

**Affiliations:** 1 Department of Conservative dentistry, Faculty of Dentistry, Cairo University, Cairo, Egypt; 2 Faculty of Dentistry, Ahram Canadian University, Giza, Egypt

**Keywords:** ICDAS II, CariescanPRO™, Digital radiography, Impedance spectroscopy, Occlusal caries, Dental equipment, Dental caries

## Abstract

The traditional methods in early caries detection had many limitations. So, this study aimed to evaluate the clinical performance of alternating current impedance spectroscopy ACIST in comparison with digital radiograph and ICDAS-II in detection of occlusal carious lesions. Occlusal surfaces of molar and premolar teeth from 40 adult participants were examined by two observers using three diagnostic methods: (1) international caries detection and assessment system (ICDAS-II) (2) digital radiograph (DR) and (3) Cariescan Pro device (ACIST). Agreement analysis and the difference in sensitivities and specificities were evaluated. The results showed an excellent agreement in the different groups. The difference from the visual tactile against ACIST scoring for enamel caries detection, was statistically significant (p = 0.012) and the agreement was moderate (k = 0.509). For dentinal caries the difference was not statistically significant (p > 0.05) and the agreement was similarly moderate (k < 0.6). The difference from the digital radiograph against ACIST scoring, for enamel caries, digital radiography had significantly higher sensitivity and specificity than ACIST (p < 0.001) while for dentinal caries detection and overall, ACIST had higher sensitivity and digital radiography had higher specificity and the difference was statistically significant (p < 0.001). Visual-tactile examination is a considered as feasible and valid technique for occlusal caries detection, digital radiography is superior to ACIST in diagnosing enamel caries, but it could underestimate the caries depth, ACIST is a reliable tool for detecting occlusal caries in dentin.

## Introduction

Dental caries is a very widespread clinical condition worldwide. According to the World Health Organisation (WHO), caries is estimated to affect 60–90% of school children and almost 90% of adults. Although dental caries remains the most widely spread chronic disease in both children and adults, it is largely preventable. According to the Global Burden of Disease Study, dental caries is considered as a one of the most common preventable non-communicable diseases globally, with an estimated 2.5 billion people affected and a 14.6% increase in dental caries over 10 years^1^. Molars and premolars are the most vulnerable teeth to caries attack related to the morphology and the difficulty of plaque removal^2^. Current evidence from epidemiological studies focusing on children, young adults, and adults have shown a shift in caries prevalence from occlusal surfaces at younger ages to proximal surfaces with advancing age. Also, the prevalence of carious lesions in adolescents and young adults remains to be high, even in populations with low risk of dental caries^3^.

Several systems for caries detection have been used in the world providing important information about the disease and guiding professionals in making treatment decisions. The most fundamental and popular technique for identifying caries in dental clinics is visual inspection. In many societies, occlusal caries beneath pits and fissures, along with clinically undiagnosed occlusal caries beneath pits and fissures, accounted for around 50% of overall caries^4^.

Combination of visual inspection (with or without probing) and radiographs are the mainstay of occlusal caries detection, but in certain cases, these procedures show low sensitivity and specificity in pits and fissure caries, and this may leave a several numbers of caries undetected^5,47^. Occlusal caries is also regarded as a significant issue in dentistry due to the challenges associated with developing a treatment plan and standardizing the diagnosis, particularly given the lesion's quiet progression and need for specialized diagnostic devices for early detection. Periapical radiographs may underestimate the depth of the carious lesion and present a very low sensitivity rate, especially in the detection of incipient caries^6^.

While radiographical examination is often used as a supplementary diagnostic tool for caries, it is important to note that clinical and radiographical assessments alone are insufficient. Periapical and bitewing radiographs are often used for clinical assessment and diagnosis. According to several reports, radiographs are more effective than clinical examination in diagnosing occlusal caries, assessing the extent of occlusal caries lesions in dentin, and monitoring changes in caries lesions^7^.

Furthermore, digital radiographs seem more efficient at detecting caries that have reached dentin than at early diagnosis of initial enamel lesions, they cannot determine whether a detected lesion is active and/or cavitated which is the threshold between operative and non-operative intervention. Also, it entails minimal but discernible risks of regularly exposing humans to ionising radiation. That’s why radiography is of especially limited value for the detection of early initial lesions, and the ionizing radiation limits its repeatability. Therefore, digital bitewing radiography is not appropriate for monitoring initial proximal lesions. Consequently, a diagnostic tool which identifies any changes in the occlusal surface rather than lesion depth is required^8^.

In recent years, adjunct methods based on fiber- optics, fluorescence or electrical impedance has been used. The first dental diagnostic device to employ the AC Impedance Spectroscopy Technique (ACIST) is the CariescanPRO™. In order to record variations in mineral density throughout the tooth, not just the surface, ACIST sends a low amplitude microamp current from the sensor tip contact through the pulp, dentin, and enamel. Enamel caries can be detected as early as possible with CariescanPRO™. This innovative tool enables prompt preventive treatment and supports evidence-based care plans by providing reliable and repeatable data to track caries over time^9^.

CariescanPRO™ uses a multiple frequency spectrum over a four-second period to measure the electrical impedance. The likelihood of carious disease is then displayed by the system, with a result ranging from 0 to 100. Together with the LED colored indicator, this numerical result gives the dentist clinician repeatable information that helps them create precise treatment plans for their patients. A tooth that is in good health typically has much higher impedance than one that has a demineralized enamel lesion, which is in turn much higher than a tooth that has established caries into the dentin. Because of this, the ACIST technology has far higher sensitivity (accurate caries detection) and specificity (accurate healthy tissue detection) than any other diagnostic technique when it comes to identifying these various stages of caries^10^.

Upon reviewing literature, it was found that previous studies focused on in-vitro research which cannot mimic the clinical situation to reach proper initial caries diagnosis^11^. Other studies focused on primary molar initial caries detection however, the current clinical trial aim to assess initial caries on the permanent molar and premolar teeth^12^. Moreover, previous studies combines many recent diagnostic tools in one study^13^ while the current study tries to concentrate on the most commonly used tools for the clinicians in their daily practice. Thus null hypothesis tested was that there is no difference in reliability of alternating current impedance spectroscopy (ACIST) compared with digital radiography and ICDAS-II in detection of occlusal carious lesions.

## Materials and methods

This diagnostic test was conducted according to standards for reporting diagnostic accuracy studies (STARD) guidelines. The different caries assessment method used in the study were, visual-tactile method (ICDAS-II), digital periapical radiograph and ACIST. All armamentariums and materials` names that were used in this study, their description, lot number, manufactures and website were illustrated in Table 1, (Supplementary Information).

**Table 1:** Tools’ names used in this study, their description, manufactures, and website.

Armamentariums or materials name	Description	Manufacture (website)
CarisanPro™^)^	Ac impedance spectroscopy technology (ACIST)	Eclipse Dental Engineering, UK https://www.duerrdental.com/
De Götzen^®^ Xgenus^®^ Dc	A wall-mounted intraoral x-ray unit	S.R.L a socio unico, Italy https://degotzen.it/en/
Siger dental sensor	A digital dental x-ray sensor	Zhuhai, China https://en.siger.cn/product/667.html
ZT dental sensor holder	A device designed to hold a digital sensor during the dental radiographic process	Tangshan Hengxin medical supply Co http://www.ztdental.com/home
Diagnostic mirror	Non magnifying mirror, front surface mouth mirror	Hu-Friedy, USA http://www.hu-friedy.com/
Community periodontal index of treatment needs (CPITN) probe	This probe has a characteristic graduated tip with a very small ball burnisher end (0.5mm) for examining carious teeth without inducing any cavitation	(HAHNENKRATT GmbH, königsbach-stein, Germany):

### Methods

#### Study setting

The present study’s protocol was registered in the protocol registration and results system (www.clinicaltrials.gov) database at (18/06/2020) with an identification number (NCT04438252). After being revised and approved by the committee of the research plan and evidence-based in the Conservative Dentistry Department, Faculty of Dentistry, Cairo University, Egypt. The procedures conducted in this research, which included human subjects, complied to the ethical criteria set by the Research Ethics Committee of the Faculty of Dentistry, Cairo University (Ethical Approval no. 20-2-10) and in accordance with the Declaration of Helsinki and its later modifications. The diagnostic clinical investigation was placed in the outpatient clinic of the conservative dentistry department at the Faculty of Dentistry, Cairo University, Egypt.

#### Study design

This is a uni-centered study on diagnostic accuracy that was designed following the STARD guidelines. The researcher had the responsibility of overseeing all operations related to the study, which included recruiting volunteers, providing them with explanations, and carrying out the procedures.

#### Sample size calculation

A power analysis was designed to have adequate power to apply statistical test of the research hypothesis that AC impedance spectroscopy will have similar diagnostic accuracy to visual inspection method using ICDAS-II and digital radiography for detection of occlusal lesions in permanent molars. According to the results of Jablonski et al.^14^ which had showed that the diagnosis accuracy of AC impedance spectroscopy was 0.84, whereas for ICDAS-II it was 0.93. By selecting a significant threshold of 0.05 (5%), the statistical power is set at 80%. The anticipated sample size (n) was 166 in total. A sample size calculation was conducted^14^. The software version is MedCalc^®^ 12.4.0 and it is developed in Mariakerke, Belgium.

#### Eligibility criteria and participant enrolment

A total of 40 participants were enrolled, and all posterior teeth were screened by both methods of diagnosis. The inclusion criteria of participants where participants age range was from 20 to 40 years with no gender restriction, having at least has one posterior occlusal suspected carious lesion, Co-operative patients who signed the informed consent, Good general health and controlled medical condition and participants with moderate or high caries risk. While for exclusion criteria of participants were presence of disabilities, rampant caries, conditions that might affect periodontal health as heavy smoking, xerostomia, pregnancy, lack of compliance, and evidence of severe bruxism, clenching, or temporomandibular joint disorders.

For inclusion criteria of the tooth to be examined was determined including posterior teeth with dark shadow through its occlusal table, vital posterior teeth with no signs of irreversible pulpitis, teeth with favorable occlusion with the opposing, non-restored teeth, and ICDAS- II scores from 0 to 4^14^. However, exclusion criteria of teeth were set as periapical pathology or signs of pulpal pathology, tooth hypersensitivity, severe periodontal affection, and lesions with ICDAS-II score 5 and 6^15^.

#### Grouping and variables of the study

The research had a total of 40 participants. Each participant had at least one probable non-cavitated occlusal lesion in either quadrant of their oral cavity (upper and lower arch) in the first and/or second molars and premolars. In all, 166 teeth were evaluated. Each lesion received evaluation using three diagnostic techniques (A), with A1 being a visual assessment method based on ICDAS-II criteria, A2 being a periapical digital radiography method, and A3 being electric impedance spectroscopy.

### Trial description

#### Recruitment strategy

The subjects’ recruitment was done by a suitable non-probability sampling method^16^. A full examination and diagnosis were done for all the participants using diagnostic dental charts. All the participants were subjected to Caries risk assessment using ADA Chart which assess the general medical and dental history of the patient, it also contains contributing conditions for dental caries. This was performed to assess the overall oral hygiene status and to evaluate each participant’s susceptibility to caries before the beginning of the investigation. Upon identification of the eligible participants for this study, they were contacted by the researcher with proper explaining of the procedure, the aim of the study, and confirming their interest to be included in the study. The interested participants must sign an informed consent which was written in a simple clear Arabic language (Supplemenary Information).

#### Randomization and assignment of ACIST, digital radiograph and ICDAS-II

No randomization was required in this trial since all patients underwent evaluation utilizing ACIST, digital radiograph and ICDAS -II assessment procedures by two examiners.

#### Blinding

The examiners were not blinded, but they were prohibited from exchanging any information for the whole research time. Prior to doing the CariScanPRO™ technique, the diagnosis using ICDAS-II was performed to prevent any bias caused by knowledge of the findings from the second method. Additionally, when the same examiner re-diagnosed the same case, the case report of the participant was concealed.

#### Examiners calibration

Both examiners have prior expertise in diagnosing using ICDAS-II criteria, interpreting digital radiographs, and using an electric impedance spectroscopy instrument. Furthermore, two weeks before the research began, calibration sessions were scheduled for the two examiners to familiarize them with the methodology. Initially, the examiners underwent training and calibration using the same methodologies that would be used in the research. Calibration was performed on a sample of 60 teeth obtained from patients at the Restorative Dentistry Clinic. Subsequently, they conducted a comparative analysis of the outcomes and closely monitored the disparities in their respective scores. Deliberation on the disparate outcomes between the two examiners continued until a final consensus was established^2^. The diagnostic techniques were overseen and standardized by field professionals.

#### Clinical examination

Participant fulfilled the inclusion criteria and enrolled in the study has undertaken the following clinical procedures: Dental prophylaxis, clinical assessment by both examiners with ICDAS-II method, clinical assessment by both examiners with (CariescanPRO™) electric impedance spectroscopy device, radiographic assessment by both examiners with periapical digital radiographic method, clinical carious lesion management of the participants according to the diagnosis score.

#### Dental prophylaxis procedures

Each participant was diagnosed by the predetermined assessment methods twice, one by each examiner (in separate cabins), and the examiners were unaware of each other’s results. The investigation sites were recorded and identified by writing the score in case report sheet^17^.

Prior to the visual examinations, scaling of all the teeth was done carefully using woodpecker^©^ ultrasonic scaler (Guilin Woodpecker Medical Instrument company, Guilin, Guangxi, China) to remove all of the surface biofilm and deposits^18^. The teeth surfaces were cleaned using prophylactic paste with low-speed prophylactic brush.

Following this preparation step of the tooth surfaces, all examinations were conducted under standardized conditions in a professional dental light with a front-surface dental mirror, lightening system, and an oil-free air syringe for drying teeth for 5 s and then isolated with cotton rolls^19^. The patient was examined twice by two examiners in the same session and the second observation was done in the recall sessions after 7 days of the first observation.

### Visual tactile examination

The patients were positioned in a reclining position in the dental unit, with an operating light illuminating the area. They were visually checked using the dental unit lamp, air–water spray, and dental mirror.

First the examiner analyzed the teeth from its occlusal surfaces after air drying of the teeth for 5 s using a triplex syringe.

The examiner was noticing any changes in translucency and color or any cavitation that indicate the state of demineralization of surface and subsurface zones compared with adjacent healthy areas. These visible signs indicating caries have been rationalized and classified using the classification system (ICDAS-II) which includes six codes^20^,Supplementary Information.

The data recorded by each examiner was written in the participant`s case report by the help of chairside assistants. The diagnosis of participant was done by visual examination in the same way and with the exact procedures of illumination, cleaning and drying to avoid any discrepancies in the recording pattern.

For ICDAS-II, the D1 threshold was calculated at code 1 as this represented the initial onset of caries into the outer half of the enamel layer. ICDAS code 2 signifies the presence of deep enamel carious lesions, the D3 threshold was calculated at both the code 3 and code 4 cutoffs, where code 3 represented an established dentinal lesion^12^.

### Digital parallel periapical radiographic examination

**Figure 1:**
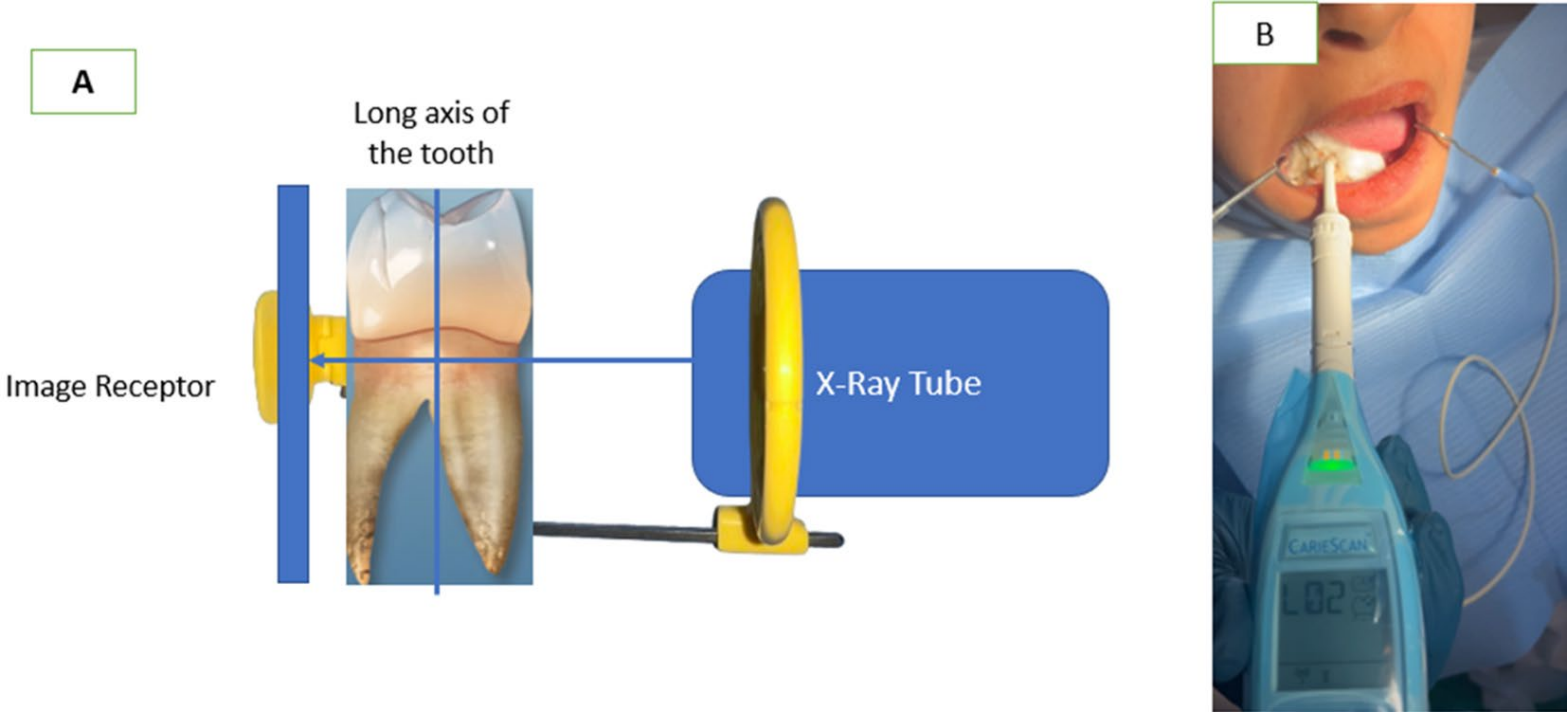
(**A**) Diagram shows the periapical parallel technique (**B**) Caries detection with impedance spectroscopy method.

Following the visual examination conducted by the examiners, the same subjects underwent an alternative evaluation approach including digital parallel periapical radiography, in this technique the periapical film is stood parallel to the long axis of the teeth and the central ray beam is aimed at the right angles of the teeth and the film (Fig. 1A), also to examine the presence of any periapical lesion during recruitment phase thus avoiding unnecessary multiple exposure to radiation in case that the patient was eligible to be enrolled in the clinical trial and fulfilling inclusions criteria. A digital x-ray sensor size 2 with dimensions 26 × 36.5 mm. (Siger dental sensor) was used in imaging after its placement in the corresponding sleeve size to allow protection against contamination of the sensor. All radiographs were taken with the parallel technique using a (Zt Dental, China) as a film holder to achieve comparable images with minimal overlap and to allow for standardization of images. The imaging plate is fixed in the bite block part of the film holder before insertion in patient mouth then the cone is directed through the aiming ring.

After exposure, the images are analyzed by the sensor software using Microsoft Window 11 PC. All the digital radiographs were primarily analyzed by the examiners. All the images were evaluated independently from all the other diagnostic findings in a darkened room with the option of adjusting the brightness and contrast by both examiners. The radiographic images were assessed and assigned scores ranging from R0 to R4 according on the criteria of the International Caries Classification and Management System (ICCMS) (Supplemenary Information).

### AC electric impedance spectroscopy examination (ACIST)

Following the visual and radiographic examinations conducted by the examiners, the same subjects underwent a further evaluation using the ACIST assessment procedure. Prior to using the CariescanPRO™, the battery underwent a minimum of 4 h of charging as per the guidelines provided by the manufacturer.

A system calibration was conducted at the start of each day and every time a sensor collar was replaced during testing. The collar that contained the sensor was taken off. The cable test adapter, which is used for system testing, was put into the neck of the CariescanPRO™ device, securely fastening it in position. The device was operated. The lip hook connection cable was inserted into the side of the device and attached to the pin of the test adapter. The test began, yielding a (OK) on the device screen on every time it was used. The cable test adapter was detached, and the collar was replaced with a new sensor**.**

Following the manufacturer’s instructions, the tooth was isolated using cotton roll and air dried using a triple syringe for 5 s and the lip hook was held at the angle of the mouth. The CariescanPRO™ sensor was placed on the tooth site to be measured and the measurement scan began as soon as the device came in direct contact with the tooth surface. To complete its scan, the sensor is held in place for approximately 4 s. The resulting value was recorded by the principal investigator (Fig. 1B). The site of interest was measured 3 times. All readings were between 0 and 100. The mean of the 3 scores was recorded. The scores are based on probability of caries with a score ranging from 0 to 50 resulting in a low probability; a score ranging from 51 to 90 resulting in a moderate probability; and a score ranging from 91 to 100 resulting in a high probability of the presence of caries in the location in contact with the device. Red, Yellow, and Green LED pyramids are illuminated on the device to correspond with the numerical score. As one would expect the green pyramid can be seen with scores from 0 to 50, the yellow with scores from 51 to 90, and red with scores from 91 to 100^21^.

Two cut-offs for D1 were taken based on manufacturer’s instructions: code 21 and code 50, corresponding to caries extension into the outer third and inner third of the enamel, respectively. The D3 threshold was calculated at cut-off 91, which represented lesion extension to the enamel-dentine junction^12^.

As a result of the difference of the scoring system in each detection method, the resulted data were categorized as shown in Table 2 to allow homogeneity in the scores and allow better statistical analysis.

**Table 2:** Different scores of each examination method and their corresponding scores in statistical analysis.

ICDAS-II scores	Digital radiography scores	ACIS scores	Corresponding scores
1	R1	ACIS1 (score 0–50)	Enamel (D1)
2	R2		
3	R3	ACIS2 (score 51–100)	Dentin (D3)
4	R4		

### Participants’ managements after diagnosis

All the participants received dental treatment for the carious teeth, according to caries score based on the previous dental findings independently of the ACIST findings and this was done for ethical, legal purposes and for patient`s benefit. Remineralization was mainly done to most of the cases as most of the lesions detected were initial and it was done by applying the MI Paste Plus, operative treatment was done for the cavitated lesions using (Filtek Z250 XT A3; 3M ESPE) restorations.

### Statistical analysis

Categorical data were presented as frequency and percentage values. Differences between measurements were analyzed using McNemar's test. Agreement analysis was done using Cohen's Kappa coefficient. The difference in sensitivities and specificities was tested using chi-square test. The significance level was set at p < 0.05 within all tests. Statistical analysis was performed with R statistical analysis software version 4.3.2 for Windows^22^.

### Ethics approval and consent to participate

The present study’s protocol was registered in the protocol registration and results system (www.clinicaltrials.gov) database, with an identification number (NCT04438252). The procedures conducted in this research, which included human subjects, complied to the ethical criteria set by the Research Ethics Committee of the Faculty of Dentistry, Cairo University (Approval no. 20-2-10) and in accordance with the Declaration of Helsinki and its later modifications. The diagnostic clinical investigation was placed in the outpatient clinic of the conservative dentistry department at the Faculty of Dentistry, Cairo University, Egypt. An informed consent with an easy Arabic language as mother language of participants was signed by the recruited participants.

## Results

### Demographic data

Summary statistics for demographic data are presented in Table 3.

**Table 3:** Summary statistics for demographic data.

Parameter		Value
Gender [n (%)] (n = 40)	Male	22 (55.0%)
	Female	18 (45.0%)
Age (mean ± SD)		35.70 ± 12.33
Arch [n (%)] (n = 166)	Upper	70 (42.2%)
	Lower	96 (57.8%)
Tooth [n (%)] (n = 166)	Premolar	66 (39.8%)
	Molar	100 (60.2%)

The study was conducted on 40 cases (i.e. 22 male and 18 females) with the mean age of (35.70 ± 12.33) (years). In which, 166 teeth were scanned (i.e. 70 upper teeth, 96 lower teeth, 66 premolars and 100 molars).

### Reliability (intra and inter-observer agreement for ICDAS-II, periapical radiograph, and CariescanPRO™)

#### Intra-observer reliability

Intra-observer reliability for different groups and observers using *Kappa* are presented in Table 4. Analysis showed there was an excellent agreement between first and second observations in different groups and for both observers.

**Table 4:** Intra-observer reliability for different groups and observers.

Group	Observer	Weighted Kappa [95% CI]
ICDAS	First	0.889 [0.811–0.962]
	Second	0.952 [0.880–0.986]
Radiograph	First	0.862 [0.761–0.900]
	Second	0.875 [0.820–0.947]
CariescanPRO™	First	0.883 [0.752–0.889]
	Second	0.850 [0.752–0.957]

#### Inter-observer reliability

Inter-observer reliability for different groups and observations are presented in Table 5. Analysis showed there was an excellent agreement between first and second observers in different groups and for both observations.

**Table 5:** Inter-observer reliability for different groups and observations.

Group	Observation	Weighted Kappa [95% CI]
ICDAS	First	0.859 [0.779–0.925]
	Second	0.952 [0.890–0.994]
Radiograph	First	0.880 [0.811–0.934]
	Second	0.840 [0.770–0.930]
CariescanPRO™	First	0.884 [0.773–0.989]
	Second	0.970 [0.896–1.025]

### Confusion matrix and differences between tests

**Figure 2:**
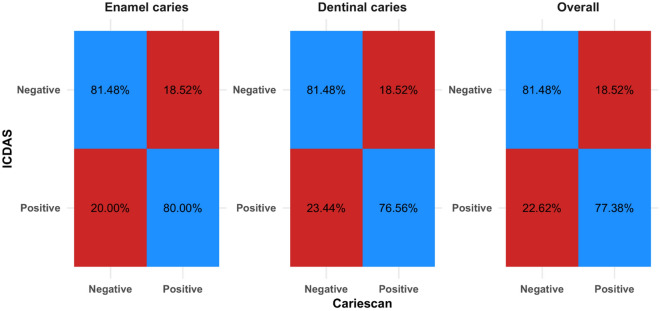
Heat map showing confusion matrix for Cariescan measurements.

Confusion matrix for Cariescan diagnosis is presented in Table 6 and in Fig. 2. For enamel caries detection, there were 19 misdiagnosed cases (i.e. 15 false positives and 4 false negatives). The difference from the ICDAS scoring was statistically significant (p = 0.012) and the agreement was moderate (k = 0.509). For dentinal caries there were 30 misdiagnosed cases (i.e. 15 false positives and false negatives). For dentinal caries and overall, the difference from ICDAS scoring was not statistically significant (p > 0.05) and the agreement was similarly moderate (k < 0.6).

**Table 6:** Confusion matrix for Cariescan measurements.

Parameter	ICDAS	CS				
		n (%)		χ^2^	p-value	Cohen’s kappa (95% CI)
		Negative	Positive			
Enamel caries	Negative	66 (81.48%)	15 (18.52%)	6.37	0.012*	0.509 (0.308:0.711)*
	Positive	4 (20.00%)	16 (80.00%)			
Dentinal caries	Negative	66 (81.48%)	15 (18.52%)	0.00	1	0.580 (0.446:0.715)*
	Positive	15 (23.44%)	49 (76.56%)			
Overall	Negative	66 (81.48%)	15 (18.52%)	0.47	0.493	0.588 (0.464:0.712)*
	Positive	19 (22.62%)	65 (77.38%)			

*CI* confidence interval, *Significant (p < 0.05).

**Figure 3:**
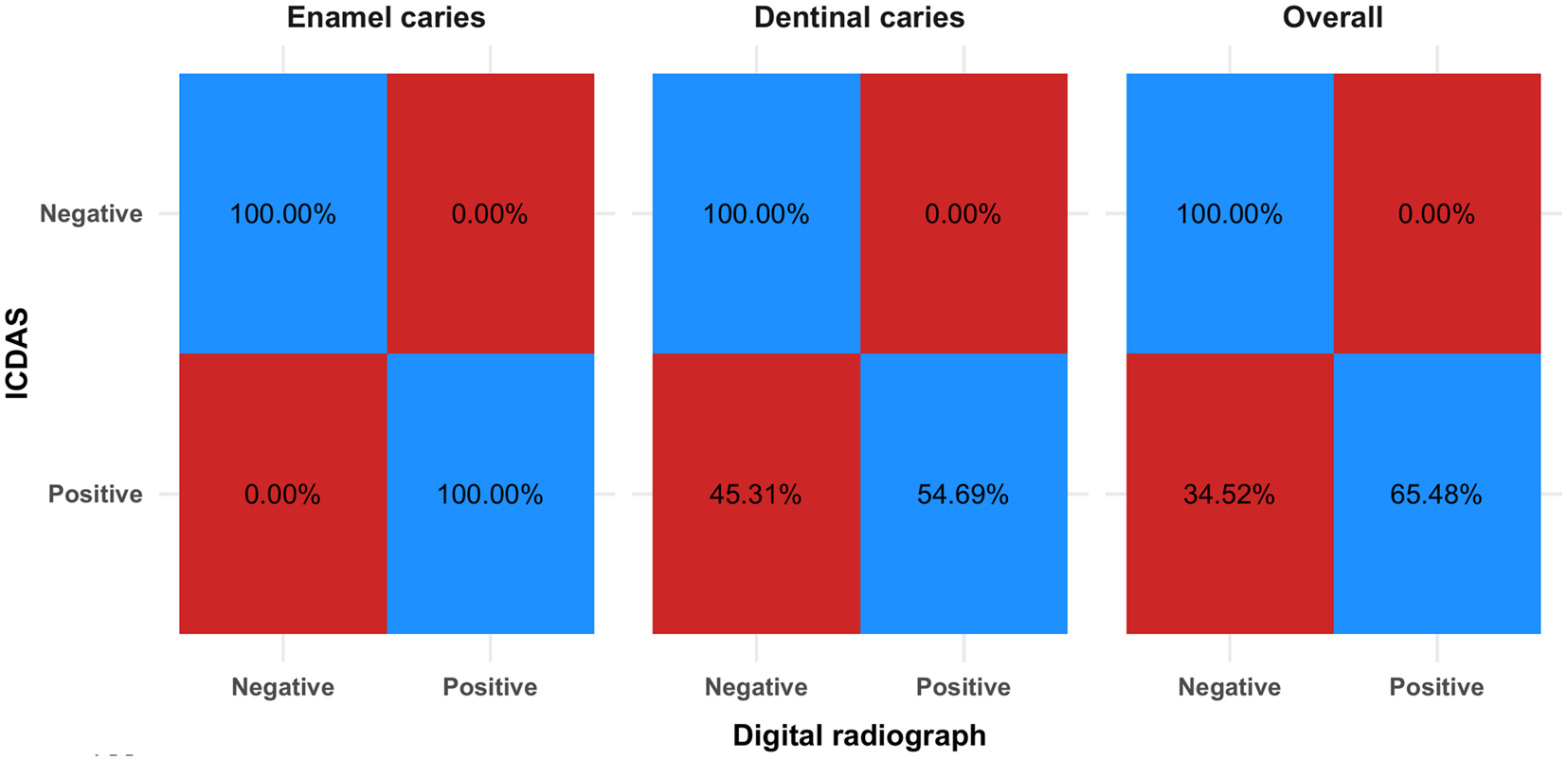
Heat map showing confusion matrix for digital radiograph measurements.

Confusion matrix for digital radiograph diagnoses is presented in Table 7 and in Fig. 3. Or enamel caries, all cases were correctly diagnosed. For dentinal caries there were 29 falsely diagnosed positive cases and the difference from ICDAS scoring was statistically significant (p < 0.001) and the agreement was moderate (k = 0.574). Overall, the difference was also statistically significant (p < 0.001), However, the agreement was substantial (k = 0.651).

**Table 7:** Confusion matrix for digital radiograph measurements.

Parameter	ICDAS	RG				
		n (%)		χ^2^	p-value	Cohen’s kappa (95% CI)
		Negative	Positive			
Enamel caries	Negative	81 (100.00%)	0 (0.00%)	NA	NA	1.000 (1.000:1.000)*
	Positive	0 (0.00%)	20 (100.00%)			
Dentinal caries	Negative	81 (100.00%)	0 (0.00%)	29.00	< 0.001*	0.574 (0.434:0.714)*
	Positive	29 (45.31%)	35 (54.69%)			
Overall	Negative	81 (100.00%)	0 (0.00%)	29.00	< 0.001*	0.651 (0.534:0.767)
	Positive	29 (34.52%)	55 (65.48%)			

*CI* Confidence Interval, *Significant (p < 0.05).

**Figure 4:**
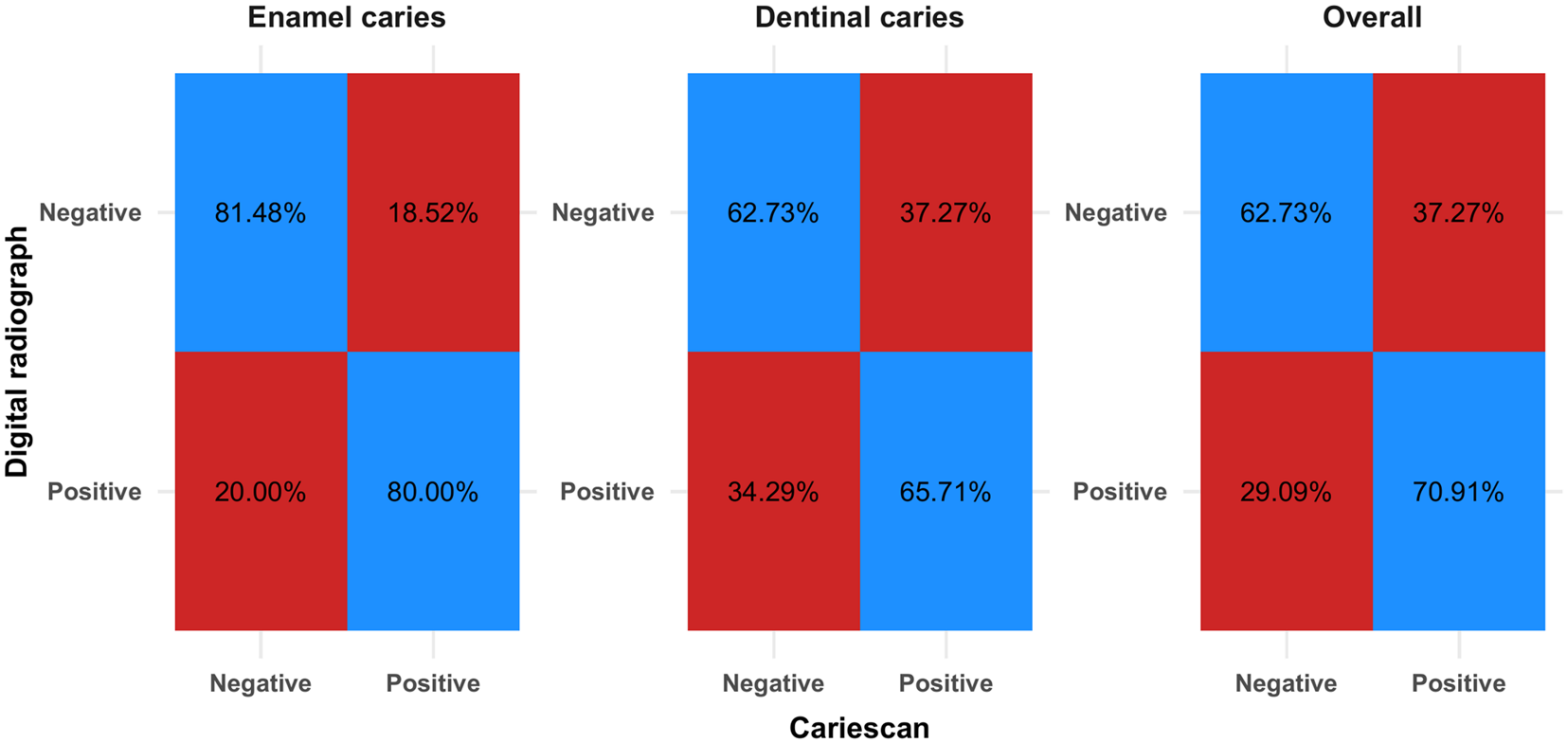
Heat map showing the agreement between tested measurements.

Differences between measurements made by tested modalities are presented in Table 8 and Fig. 4. For enamel caries there was disagreement in the diagnosis of 19 cases, the difference was statistically significant (p = 0.012) and the agreement was moderate (k = 0.509). For dentinal caries, there was a disagreement in the diagnoses of 53 cases, the difference (i.e. for dentinal caries and overall) was statistically significant (p < 0.001) and the agreement was fair (k < 0.4).

**Table 8:** Difference between tested measurements.

Parameter	RG	CS				
		n (%)		χ^2^	p-value	Cohen’s kappa (95% CI)
		Negative	Positive			
Enamel caries	Negative	66 (81.48%)	15 (18.52%)	6.37	0.012*	0.509 (0.308:0.711)*
	Positive	4 (20.00%)	16 (80.00%)			
Dentinal caries	Negative	69 (62.73%)	41 (37.27%)	15.87	< 0.001*	0.222 (0.053:0.390)*
	Positive	12 (34.29%)	23 (65.71%)			
Overall	Negative	69 (62.73%)	41 (37.27%)	10.96	< 0.001*	0.302 (0.154:0.450)*
	Positive	16 (29.09%)	39 (70.91%)			

*CI* confidence interval, *Significant (p < 0.05).

Difference in accuracy of tested modalities is presented in Table 9. For enamel caries, digital radiography had significantly higher sensitivity and specificity than Cariescan (p < 0.001). For dentinal caries detection and overall, Cariescan had higher sensitivity and digital radiography had higher specificity and the difference was statistically significant (p < 0.001).

**Table 9:** Difference in accuracy between tested modalities.

Parameter	Parameter	Value (95% CI)		χ^2^	p-value
		Cariescan	Digital radiography		
Enamel caries	Sensitivity	80.00% (56.34%:94.27%)	100.00% (83.16%:100.00%)	19.00	< 0.001*
	Specificity	81.48% (71.30%:89.25%)	100.00% (95.55%:100.00%)		
	PPV	51.61% (33.06%:69.85%)	100.00% (83.16%:100.00%)		
	NPV	94.29% (86.01%:98.42%)	100.00% (95.55%:100.00%)		
	PLR	4.32 (2.60:7.17)	Inf		
	NLR	0.25 (0.10:0.59)	0.00 (0.00: 0.00)		
	Accuracy	81.19% (72.19%:88.28%)	100.00% (96.41%:100.00%)		
Dentinal caries	Sensitivity	76.56% (64.31%:86.25%)	54.69% (41.75%:67.18%)	20.16	< 0.001*
	Specificity	81.48% (71.30%:89.25%)	100.00% (95.55%:100.00%)		
	PPV	76.56% (64.31%:86.25%)	100.00% (90.00%:100.00%)		
	NPV	81.48% (71.30%:89.25%)	73.64% (64.38%:81.58%)		
	PLR	4.13 (2.57:6.66)	Inf		
	NLR	0.29 (0.18:0.45)	0.45 (0.35:0.59)		
	Accuracy	79.31% (71.80%:85.58%)	80.00% (72.56%:86.18%)		
Overall	Sensitivity	77.38% (66.95%:85.80%)	65.48% (54.31%:75.52%)	17.38	< 0.001*
	Specificity	81.48% (71.30%:89.25%)	100.00% (95.55%:100.00%)		
	PPV	81.25% (70.97%:89.11%)	100.00% (93.51%:100.00%)		
	NPV	77.65% (67.31%:85.97%)	73.64% (64.38%:81.58%)		
	PLR	4.18 (2.61:6.69)	Inf		
	NLR	0.28 (0.18:0.42)	0.35 (0.26:0.46)		
	Accuracy	79.39% (72.41%:85.29%)	82.42% (75.74%:87.90%)		

*CI* confidence interval, *Significant (p <0.05).

### Representative radiographs and clinical photos

**Figure 5:**
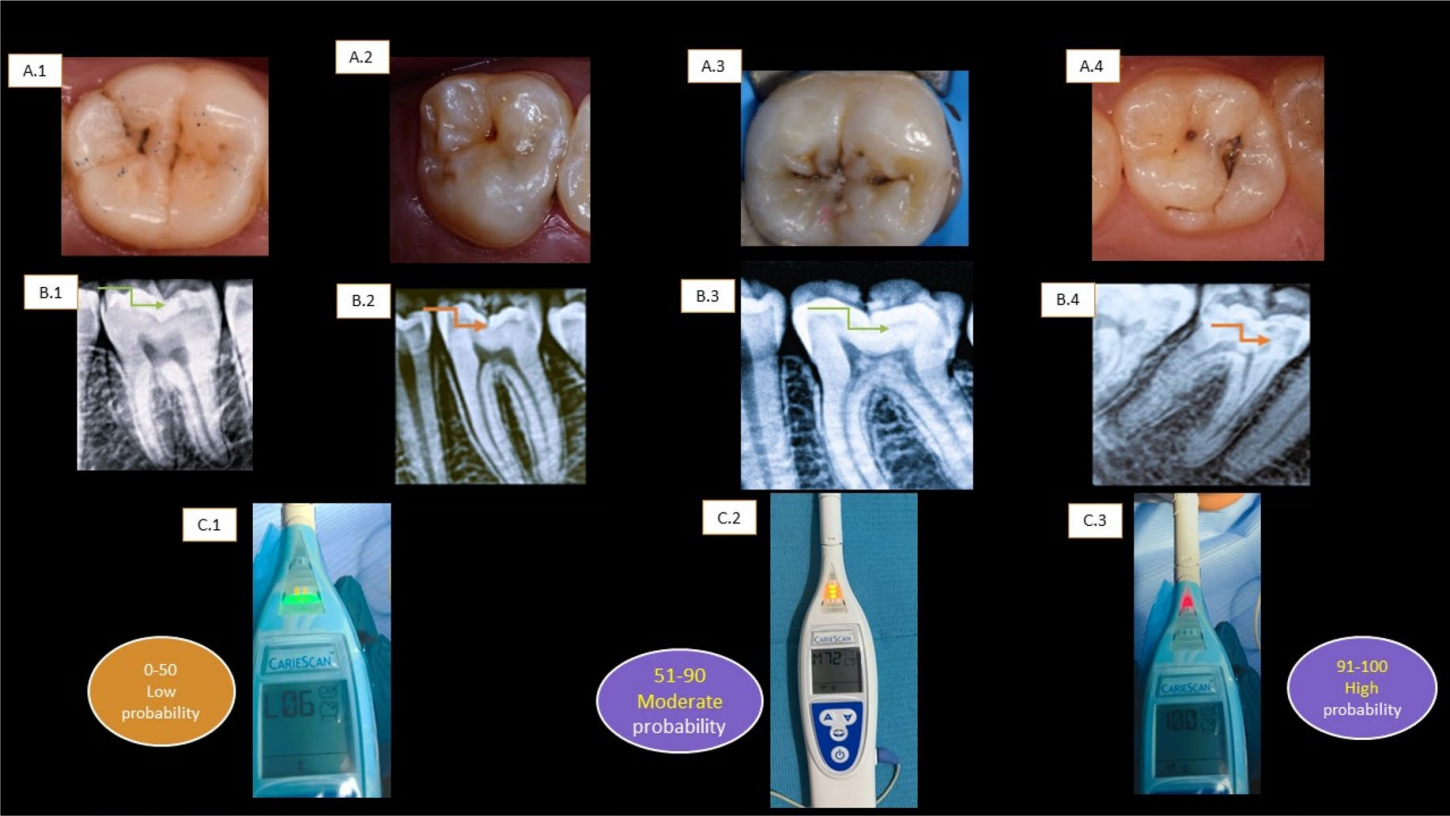
Shows different ICDAS-II scores (**A**) with its corresponding periapical digital radiographs (**B**) and CariesScan Pro scores (**C**). (**A.1**) Clinical Photo representing ICDAS score 1. (**A.2**) Clinical Photo representing ICDAS score 2. (**A.3**) Clinical Photo representing ICDAS score 3. (**A.4**) Clinical Photo representing ICDAS score 4. (**B.1**) Digital periapical radiograph showing occlusal caries in lower molar in the outer half of enamel with score R1 (red arrows). (**B.2**) Digit al periapical radiograph showing occlusal caries in lower molar in the inner half of enamel with score R2 (red arrows). (**B.3**) Digital periapical radiograph showing occlusal caries in lower molar in outer third of dentin with score R3 (red arrows). (**B.4**) Digital periapical radiograph showing occlusal caries in lower molar in middle third of dentin with score R4 (red arrows) dentin with score R4 (red arrows). (**C.1**) scores 0–50 with green led light resulting in a low probability, (**C.2**) a score ranging from 51 to 90 with yellow led light resulting in a moderate probability, (**C.3**) a score ranging from 91 to 100 with red led light resulting in a high probability of the presence of caries.

Clinical photos which show different ICDAS-II scores (A1-A4)) with its corresponding periapical digital radiographs (B1-B4)) and CariesScan Pro scores(C1-C3) (Fig. 5).

## Discussion

Screening of dental caries lesions is considered as one of the most frequent tasks in general dental practice, aiming to detect early carious lesions to provide non- or microinvasive treatments and consequently prevent more invasive and expensive restorative cycles. The occlusal and proximal teeth surfaces are commonly susceptible to dental caries, which are usually developed in areas of stagnation where plaque could accumulate undisturbed^21^*.*

Detecting non-cavitated early lesions is challenging even for highly skilled doctors. Accurate evaluation allows for the use of suitable preventative and regenerative therapies, while inaccurate evaluation frequently results in unneeded restorative treatment, which adds to the patient’s financial burden and condemns the tooth to repeated restoration in the future^23^. From a clinical perspective, visual and tactile examination is still considered the most reliable method for detecting and diagnosing caries. Nevertheless, tactile examination is characterized by a low level of prediction accuracy. However, despite the American Dental Association's advice to refrain from using sharp explorers, a significant number of practitioners still largely depend on the "catch" as evidence of caries activity, even after almost 25 years. The absence of viable alternative diagnostic techniques has unquestionably prolonged this predicament^24,25^.

In this study occlusal caries was selected as occlusal surfaces are frequently afflicted by dental caries, with lesion detection being challenging for these surfaces Given the potential costs emanating from both controlling and managing these surfaces, so emerging technologies have been developed for reliable occlusal caries detection^26,27^.

The primary objective of contemporary dentistry is to immediately identify first carious lesions. Nevertheless, the identification of dental caries has consistently posed challenges. The identification of non-overt occlusal decay is a difficult task and can be extremely subjective, with inherent ambiguities that might result in significantly divergent treatment choices. Enhancing the sensitivity, specificity, and reproducibility of diagnostic instruments for occlusal surfaces would significantly enhance the accuracy of preventive and surgical therapy planning^28^.

The international caries detection and assessment system (ICDAS) is a clinical scoring system that utilizes visual inspection, with the option of using a ball-ended probe, to aid in the detection of occlusal caries. This system is particularly useful for identifying surface texture and removing debris in a gentle manner. The approach categorizes different stages of the caries process by considering the histological extent and activity. Hence, the tooth surface is visually examined to distinguish seven stages ranging from "sound" to "extensive distinct cavity with visible dentin." These steps are intended to correspond with the depth of the cavity and, thus, aid in determining an appropriate treatment plan. The capacity to identify and categorize caries lesions in their first stages has a substantial influence on treatment choices and can enhance the likelihood of a successful preventative intervention^29,30^.

However, there are several problems encountered with visual tactile interpretation for occlusal caries, according to Featherston et al.^31^ the diagnosis of occlusal caries is a complex process that requires considering multiple factors, rather than a straightforward yes or no answer. To understand a color shift on the surface, it is necessary to assess both external and internal factors. It is necessary to differentiate between opacities caused by enamel developmental abnormalities or fluorosis and those caused by demineralization and caries. Hence, the presence of coarse enamel formations suggests ongoing dental deterioration^.^ In addition, the dry tooth surface can be assessed for its level of chalkiness. If there is any doubt regarding the presence of cavitation on the surface, a ball-ended probe can be employed to delicately examine the surface texture and determine if there is any discontinuity or fracture. A differentiated caries diagnosis is necessary due to the discovery that non-cavitated lesions have the potential to undergo remineralization without causing any damage to the tooth, as long as the crystalline structure remains intact.

On the other hand, digital periapical radiography is frequently used to determine caries depth extending into the dentine^32,33^ (however periapical radiographs may underestimate the depth of the carious lesion and present a very low sensitivity rate, especially in the detection of incipient caries^30^. The incorporation of both visual and radiographic examination enhances the sensitivity of the standalone visual check. Nevertheless, employing a concurrent approach in techniques leads to an escalation in the occurrence of inaccurate positive outcomes, so diminishing the distinctiveness of each individual method. This phenomenon results in a rise in the quantity of superfluous interventions. Also, there are some concerns regarding the exposure of patients to ionizing radiation. In accordance with the principle of “as low as reasonably achievable”, which involves keeping to a minimum the amount of radiation that patients can be exposed to, the X-rays are only indicated when they are necessary^34^*.*

The limitations of traditional caries detection methods and advancements in technology have resulted in the creation of contemporary equipment for early caries detection. From these devices are the alternating current impedance spectroscopy based devices. Of the alternating current spectroscopy impedance is a contemporary technique for detecting caries that does not require intrusive procedures. The idea relies on the fact that undamaged enamel functions as an effective insulator in an electrical circuit due to its chemical composition. The occurrence undergoes a transformation when the solid tooth structure is damaged by tooth decay, allowing the measurement and quantification of the demineralization of the enamel or dentin using ACIST.

The CariescanPRO™ devices (Cariescan Ltd., Dundee, UK) is the current commercially available system for detecting smooth surfaces and cracks using ACIST technology. During the examination, several frequencies of alternating currents are applied to the tooth, resulting in the generation of a spectrum of impedance points. The measurement result is analyzed by the software embedded in the device and shown both numerically on a scale ranging from 0 to 100 and through color coding, ranging from green to red. The severity of the lesion's spread increases proportionally with increasing number values^35^*.*

CariescanPRO™ provides an objective depiction of caries-related alterations in the hard tooth structure to the patient. With the growing emphasis on prevention in dentistry, effective communication with patients and enhanced follow-up procedures are becoming increasingly important. By utilizing numerical lesion quantification, it becomes feasible to initially examine the teeth and implement a demineralizing intervention as a form of "monitoring," but to proceed with invasive measures only if there is evidence of a progressive carious alteration^36^.

The goal behind the production of the Cariescan Pro according to the manufacturer is to enhance the detection of caries even in initial phases of development with no need for tooth exposure. It seems to be a safe technology for caries detection compared to radiography^12^*.*

A few studies were conducted to assess the the validity and accuracy of alternating current impedance spectroscopy devices^37,38^*.* Thus, this study was conducted to assess the accuracy of Alternating Current Impedance Spectroscopy device (CariescanPRO™) in comparison with digital radiographic findings and visual examination using ICDAS-II in detection of occlusal carious lesions.

Nevertheless, the human eye (and brain) remains an essential foundation in any process. However, the proficiency of a clinician is influenced by numerous factors, and two doctors with varying experiences may provide disparate interpretations or evaluations. The heterogeneity in perception and interpretation is a significant challenge in clinical decision-making. It is advisable to employ multiple observers and conduct independent readings to have a comprehensive understanding of the potential fluctuations in a specific diagnostic test^39^*.*

The null hypothesis tested in the current study was partially accepted regarding comparison between alternating current impedance spectroscopy (CariescanPRO™) and visual tactile method (ICDAS-II) as there was no statistical significance difference between the two tested method, while it was partially rejected regarding comparison between electric impedance spectroscopy and digital radiographic assessment method in detection of occlusal carious lesions.

The results of inter and inter observer agreement in the current study was calculated by Kappa test and they showed an excellent agreement in the different groups (Tables 4, 5). These results may be due to the calibration sessions which were done for both examiners regarding the optimal use of the three diagnostic methods. Such calibration was done clinically to avoid any change in the surrounding diagnostic circumstances. Furthermore, the implementation of standardised diagnostic procedures contributed to the achievement of a high level of agreement. This was facilitated using identical calibrated screens and lighting conditions by the examiners during their evaluations. Moreover, the results showed higher kappa values for inter examiner agreements. This may be explained by the fact that both observers obtained more experience in the evaluation methods. These results agree with Şenel et al. 2010^40^ who found very high interobserver kappa coefficients that suggest excellent and strong interobserver agreement among experienced observers for all diagnostic methods with interobserver kappa coefficients ranged from 0.631 to 0.811.

Current results showed that there was a significant difference and a moderate statistically significant agreement between different modalities (Tables 6 and 7). The differences between measurements made by tested modalities presented in Table 8 and Fig. 4 revealed that for enamel caries there was disagreement in the diagnosis of 19 cases, the difference was statistically significant (p = 0.012) and the agreement was moderate (k = 0.509). For dentinal caries, there was a disagreement in the diagnoses of 53 cases, the difference (i.e. for dentinal caries and overall) was statistically significant (p < 0.001) and the agreement was fair (k < 0.4).

According to Tencate et al.^41^, CariescanPRO™ demonstrated higher accuracy levels at D3 cutoff levels, but reduced diagnostic accuracy compared to visual inspection at the D1 threshold. The underperformance of CariescanPRO™ at the dentin-enamel junction (DEJ) may be attributed to the manufacturer’s narrow threshold measurements. Furthermore, it is widely recognized that DEJ has a lower mineral content compared to both enamel and dentin, which possess a greater amount of organic matrix. As a result, the AC-impedance reading of CariescanPRO™ was significantly lower than anticipated.

Furthermore, the findings of the present investigation were consistent with those of Popuri et al.^42^ who reported that CariescanPRO™ demonstrated a considerably greater diagnostic accuracy at dentine (D3) compared to the other two modalities (p < 0.001). Therefore, it suggests that CariescanPRO™ shown superior performance in accurately determining the extent of caries at deeper layers, and this performance is partly influenced by the threshold values used to categorize the numerical output into scores for sound teeth, enamel caries, or dentine caries. Accuracy is the crucial determinant for predicting the effectiveness of diagnosis.

Pitts et al.^43^ suggested that CariescanPRO™ may have a reasonable performance at detecting demineralized tissue at the enamel cutoff level. This is because it can identify demineralization that is not visible with traditional examinations and operational intervention (considered the gold standard). The CariescanPRO™ is a reliable diagnostic tool for detecting advanced stages of tooth decay and is particularly effective at identifying enamel caries. The data indicate that the overall performance of CariescanPRO™ is satisfactory.

Hamilton et al.^44^ stated that the possible explanation for lower accuracy of visual examination in the present study can be attributed to the fact that, in visual examination, many lesions are left undetected due to the macroscopically intact surface (hidden caries) or wrongly diagnosed as enamel caries, thus allowing the dentinal lesions to progress unchecked. In addition, dental caries is a dynamic process in which early lesions undergo demineralization before being expressed clinically, thus being missed visually.

There was statistically significant difference in the score distribution in both CariescanPRO™ and digital radiography modalities (Table 9). For enamel caries, digital radiography had significantly higher sensitivity and specificity than Cariescan (p < 0.001). For dentinal caries detection and overall, Cariescan had higher sensitivity and digital radiography had higher specificity and the difference was statistically significant (p < 0.001). These findings might be referred to the underestimation of enamel lesions by the digital radiograph as at least 30% of enamel demineralization is necessary to be detected radiographically^45^.

Furthermore, the current study demonstrated a statistically significant difference in the ability to detect occlusal caries when comparing to visual examination, digital radiograph, and CariescanPRO™. When there was a stain on the occlusal surface, only the CariescanPRO™ was capable of accurately determining the severity of tooth decay, whereas visual examination and digital radiography were unable to do so. In such instances, the determination of treatment is entirely reliant on CariescanPRO™.

Mestriner et al.^46^ found that visual inspection is an important diagnostic method; conventional bitewing and digital radiography aid the diagnosis and are equally efficient to diagnose carious lesions in the dentine of teeth without visible cavitation, also Melo et al. 2022^47^ established a clinical protocol for detecting occlusal caries, which suggest using the ICDAS-II visual method as the initial approach (although it only detects 38.4% of the lesions). If CariescanPRO™ fails to successfully identify occlusal caries, digital radiography should be used instead to minimize patient exposure to ionizing radiation. This is especially important for children and pregnant women. For instance, in the context of cancer diagnosis, the screening process has mostly targeted groups with a higher likelihood of developing the disease^48^.

### Limitations of the study

Since the study involved molars and premolars, it will be challenging to create a dry field for caries detection. The presence of saliva on the carious lesion affects the readings of the CariescanPRO™, resulting in higher readings and a higher level of caries. Tooth position either upper or lower or type of tooth (molar, premolar) or accessibility of the tooth, all these challenges should be taken into consideration for each diagnostic test.

## Conclusions

Under the conditions of the current study the following conclusions might be evident.

1. Visual tactile examination is considered gold standard for occlusal caries detection.
2. Digital radiography is considered an adjunct diagnostic aid for caries detection, but it underestimates the caries depth.
3. CariescanPRO™ is a reliable and accurate diagnostic tool that can complement a visual exam for detecting occlusal caries.
4. CariescanPRO™ is an accurate diagnostic method especially in detection of dentin lesions.

### Recommendations

1. The effect of ACIST diagnostic findings should be assessed in terms of cost-effectiveness, effect on treatment decisions and its benefits as a screening tool in daily dental practice.
2. More studies are needed to assess efficacy of CariescanPRO™ with standardized methodologies to allow for reaching reliable conclusion validating its application in daily dental practice.

### Supplementary Information

Supplementary Information.

## Data Availability

The data that support the findings of this study are available from the corresponding author, on reasonable request.

## Abbreviations

ACIST: Alternating current impedance spectroscopy technology (CariescanPRO™)
ICDAS: International caries detection and assessment system
DR: Digital radiography
ROC: Receiver operating characteristics
WHO: World Health Organization
LED: Light emitting diode
