# Supplementary material for: Reliability of impedance spectroscopy versus digital radiograph and ICDAS-II in occlusal caries detection: a prospective clinical trial
Source: Sci Rep. 2024 Jul 17;14:16553. doi: 10.1038/s41598-024-66627-4 (PMC11255227; doi:10.1038/s41598-024-66627-4)

**Reliability of Impedance Spectroscopy versus digital radiograph and ICDAS-II in occlusal caries detection: A prospective clinical trial**

**Armamentarium descriptions:**

**AC Impedance Spectroscopy (CariScan Pro ^TM)^) (ACIST) (Eclipse Dental Engineering, UK):**

The System includes: Cariescan PRO™, Lip Hook Cable, Cable Test Adaptor, Power Supply Unit, Charging Cradle, Sensors, and Collars.

**Intraoral X-ray unit:(** De Götzen**® DC** S.r.l. a socio unico**, italy):**

It is a wall-mounted intraoral x-ray unit with High-frequency generator for constant high voltage**,** Tube voltage: 70 kV**,** Tube current: 8 mA**,** Focal spot: 0.4 (IEC 336)**,** Line voltage: 120–240 V ± 10 % without switching, 50/60 Hz.**,** Exposure time: Incrementally adjustable from 0.01 to 3.2 s**,** Detector media: Pre-set for film, phosphor plates and digital sensor systems, Display: Multi-colored display to show the different system states and Focus-to-skin distance: Standard 20 cm, optional 30 cm

**Digital dental sensor (Siger dental Sensor) (Zhuhai, China):**

A digital sensor that can be operated with the Siger software using Window operating system, the Digital sensor features are sensor sizes was 2 (active area: 26*36.5 mm), Pixel Number: 1000*1500, Cable Length: 3 meters, Resolution: 25-line pairs per millimeters, Dimensions ((H x W x D mm)): 226 x 234 x 243, CMOS type dental sensor and USB Interfaces. A Sensor holder (ZT Dental, China) was used during the dental radiographic process. This device has 3 components: bite-blocks, indicator arm and aiming ring. Bite-blocks used to hold the film or image pate intraorally. Indicator arm connects the bite-block with the aiming ring. The aiming ring is used in standardization and placement of the x-ray device cone. ZT Dental system is provided with different color coding for different radiographic techniques. The red one was used in this study as it was the color corresponding to the selected radiographic technique.

**High definition dental mirror (Hu-Friedy,Chicago, USA):**

It was a front surface dental mirror which makes the visual examination more precise and clearer. It provides a high degree of light reflection (113%) and provides excellent indirect vision for examination of patients’ teeth. It is brighter than other types of front surface mirrors by about 38.5% -50%. Also, it has a scratch-resistant surface for product longevity. Its ergonomic handle increases control, reduces hand fatigue, and provides maximum comfort in usage.

**Community Periodontal Index of Treatment Needs (CPITN) probe (HAHNENKRATT GmbH, königsbach-stein,Germany):**

This probe has a characteristic graduated tip with a very small ball burnisher end (0.5mm) for examining carious teeth without inducing any cavitation.

**the classification system (ICDAS-II) which includes six codes:**

| **Score 0:** | Sound tooth surface: No evidence of caries after 5 sec air drying |
| --- | --- |
| **Score 1:** | First visual change in enamel: Opacity or discoloration (white or brown) is visible at the entrance to the pit or fissure seen after prolonged air drying |
| **Score 2:** | Distinct visual change in enamel visible when wet, lesion must be visible when dry |
| **Score 3:** | Localized enamel breakdown (without clinical visual signs of dentinal involvement) seen when wet and after prolonged drying |
| **Score 4:** | Underlying dark shadow from dentine |
| **Score 5:** | Distinct cavity with visible dentine |
| **Score 6:** | Extensive (more than half the surface) distinct cavity with visible dentine |

**The radiographic images were assessed and assigned scores ranging from R0 to R4 according on the criteria of the International Caries Classification and Management System (ICCMS):**

R0: no radiolucency

R1: radiolucency in the outer half of the enamel

R2: radiolucency in the inner half of the enamel with or without reaching the dentino-enamel junction.

R3: radiolucency limited to the outer third of the dentin.

R4: radiolucency reaching the middle third of the dentin.

**ADA chart**


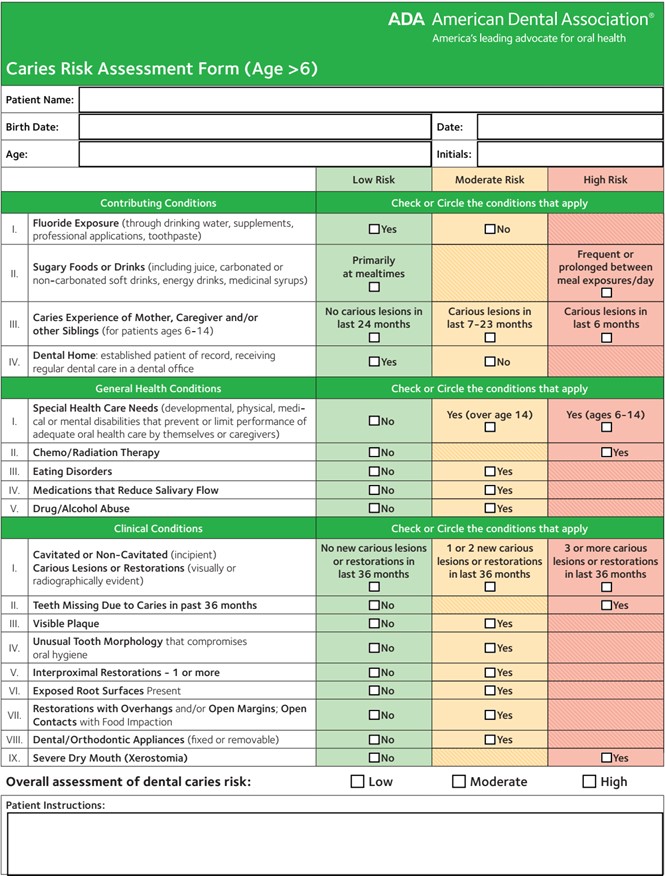

Supplement: Supplementary file 1 — Supplementary Information. [file 41598_2024_66627_MOESM1_ESM.docx]
